# Predictive Value of *Kozak* Gene Polymorphism for Thrombosis in Patients with Philadelphia-Negative MPNs

**DOI:** 10.31557/APJCP.2021.22.4.1195

**Published:** 2021-04

**Authors:** Mohamed Sabry El-Ghonemy, Shaimaa El-Ashwah, May Denewer, Eman Adel Soliman, Mohammed El-Baiomy, Heidi Elkerdawy, Ahmed EL-Sebaie

**Affiliations:** 1 *Hematology Unit, Department of Clinical Pathology, Faculty of Medicine, Mansoura University, Mansoura, Egypt. *; 2 *Clinical Hematology Unit, Oncology Center Mansoura University, Faculty of Medicine, Mansoura, Egypt. *; 3 *Specialist at Molecular Biology Lab., Oncology Center Mansoura University, Mansoura, Egypt. *; 4 *Medical Oncology Unit, Oncology Center Mansoura University, Faculty of Medicine, Mansoura, Egypt. *; 5 *Clinical Hematology Unit, Internal Medicine department, Faculty of Medicine, Port Said University, Port Said, Egypt. *

**Keywords:** Kozak, MPNs, polycythemia, thrombocythemia, myelofibrosis

## Abstract

**Background:**

Philadelphia-negative myeloproliferative neoplasms (MPNs) including polycythemia vera (PV), essential thrombocythemia (ET) and myelofibrosis are clonal haematopoietic stem cell disorders characterized by dysregulated proliferation. The arterial and venous thromboses are the major causes of morbidity and mortality in MPNs. The platelet GP Ib-IX-V receptor complex plays an important role in thrombus formation as the *Kozak* sequence polymorphism of platelet GP Ibα is associated with increased receptor density.

**Materials and Methods:**

This study was conducted on 286 diagnosed patients with Ph-negative MPNs (94 patients of PV, 102 of ET and 90 of MF). In addition, 107 apparently healthy individuals served as a control group.

**Results:**

This study revealed that by taking rs2243093 TT as the reference genotype and T as the reference allele; TC, CC, TC+CC genotypes showed lower frequency in ET patients (p= 0.005, 0.007 and 0.001 respectively) and MF patients (p= 0.002, 0.047 and 0.001 respectively) when compared to control groups also, C allele in both groups compared to control (p ≤ 0.001 both). CC genotypes and C allele showed lower frequency in PV patients when compared to control groups (p= 0.032 and 0.026 respectively).

**Conclusion:**

From this study we could conclude that patients with Philadelphia-negative MPNs carried *Kozak* gene polymorphism significantly TT genotype in all patients PV, ET, MF patients and TC in ET and MF patients. The platelet glycoprotein Ibα (*Kozak*) gene could be incorporated into the routine workup to predict venous thrombosis in patients with Ph-negative MPNs specially ET patients.

## Introduction

The myeloproliferative neoplasms (MPNs) are clonal haematopoietic stem cell disorders that are characterized by dysregulated proliferation of one or more cell lineages, resulting in over production of granulocytes, red cells, megakaryocytes, mast cells or a combination of these lineages (Barbui et al., 2013). 

The World Health Organization (WHO) classification system for hematopoietic tumors was recently revised and the 2016 document recognizes myeloproliferative neoplasms (MPNs) as one of several myeloid malignancies. Philadelphia-negative MPNs is an operational sub-category of MPNs that includes polycythemia vera (PV), essential thrombocythemia (ET), primary myelofibrosis (PMF) and prefibrotic primary myelofibrosis (prePMF) while Philadelphia-positive MPNs include chronic myeloid leukemia (CML) (Barbui et al., 2016). 

These clonal myeloproliferative disorders of are characterized by mutually exclusive *JAK2, CALR*, and MPL mutations. Almost all patients with PV harbor a *JAK2* (Janus kinase 2; 9p24) mutation; approximately 96% and 3% displaying somatic activating mutations in exon 14 (JAK2V617F) and exon 12 of JAK2, respectively. JAK2V617F also occurs in ET and PMF, including prePMF, with respective mutational frequencies of 55% and 65%. JAK2 exon 12 mutations are rare in ET or PMF. Calreticulin (CALR: 19p13.2) mutations are rare in PV but occur in 25%-35% of patients with PMF and 15%-24% with ET. CALR is a multi-functional Ca21 binding protein chaperone mostly localized in the endoplasmic reticulum (Vannucchi et al., 2008; Tefferi et al., 2014).

Life expectancy of patients with MPNs particularly that of subjects with polycythemia vera (PV) and essential thrombocythemia (ET) has significantly increased due to the use of cytoreductive treatments. Polycythemia vera and essential thrombocythemia are characterized by increased risk of thrombosis that occurs in about 20% of patients at diagnosis and considered a major cause of morbidity and mortality in these patients, so the main goal of treatment is to prevent thrombotic complications (Gangaraju et al., 2020).

The pathogenesis of thrombosis in MPNs is multifactorial so it has been extensively investigated by focusing in particular on the possible contribution of disease related haemostatic abnormalities. Quantitative and qualitative red blood cell, platelet, and leukocyte abnormalities are likely to play a key-role in myeloproliferative neoplasm thrombophilia (Landolfi and Di Gennaro, 2011)

Platelets have an essential role in thrombosis and haemostasis. Platelets adhere to the exposed sub-endothelial components as collagens or laminins, forming a plug, if this process is uncontrolled it may lead to thrombotic events. Platelet adhesion and activation is a multistep process which involves multiple platelet receptor-ligand interactions between the glycoprotein (GP) Ib-IX-V complex and von Willebrand factor (VWF) bound to collagen (Niewswandt et al., 2011).

Glycoprotein Ib-IX-V is a platelet membrane receptor complex consists of 4 polypeptides; GPIbα, GPIbB, GP IX, and GP V, which plays a main role in mediating platelet activity and thrombosis. The largest subunit is GPIbα which contains all of the known extra cellular ligand-binding sites. It also contains the binding site for von Willebrand factor (VWF) (Andrews et al., 2003)

There are four polymorphisms associated with GPIbα; the human platelet antigen-2 (HPA-2) system, tandem repeats (VNTR) in the macroglycopeptide region, the Kozak sequence which contains a single nucleotide substitution and Taq polymorphism in the 3` untranslated regions (Frank et al., 2001).

The *GPIbα* gene (*Kozak*) sequence polymorphism is the result of either a T (thymine) or a C (cytosine) at position −5 relative to the ATG start codon. It was observed that the close proximity of C allele to the kozak consensus sequence leads to increased expression of GPIb-IX-V complex on the platelet surface (Yonal et al., 2012). Many studies on the Kozak genotype frequency and haplotype analysis confirmed the association between *Kozak* polymorphism and thrombosis. This study aimed to evaluate the incidence and the predictive value of *Kozak* gene polymorphism for thrombosis in patients with Philadelphia-negative MPNs.

## Materials and Methods

This study was conducted on 286 patients with Philadelphia-negative MPNs, after approval of the Local Ethics Committee of Mansoura University and obtaining written informed consent from all patients, admitted to the Mansoura Oncology Center (94 PV patients, 102 ET patients and 90 MF patients). In addition, 107 apparently healthy individuals were served as a control group. The following parameters were carried out to all patients; full history taking, complete clinical examination, laboratory investigations (routine, BMA and molecular investigation in the form of *JAK-2 V617F* mutation and calreticuline mutation) and radiological investigation by color Doppler ultrasonography to follow up the conducted patients for 24 months to detect lower limbs and upper limbs, portal and mesenteric venous thrombosis also arterial thrombosis. MPNs were diagnosed on the basis of criteria established by the World Health Organization (Arber et al., 2016).

*JAK-2 V617F mutation using ASO specific PCR Genetic marker*

The detection of jak2 V617F mutation were done by using the following primers (JAK2 Reverse: 5’ CTGAATAGTCCTACAGTGTTTTCAGTTTCA 3’, JAK2 Forward (specific): 5’ AGCATTTGGTTTTAAATTATGGAGTATATT 3’ and JAK2 Forward (internal control): 5’ ATCTATAGTCATGCTGAAAGTAGGAGAAAG 3’). Cycling conditions were 35 cycles with annealing temperature 58.5oC flowed by 2% agarose gel electrophoresis for bands detections. For positive samples, the V617F burden allele was done by the real-time PCR method (Larsen et al., 2007).

*Calreticuline mutation screen*

The following primers (CALR Forward: 5’ TAACAAAGGTGAGGCCTGGT 3’ and CALR reverse: 5’ GCCTCTCTACAGCTCGTCCTT 3’). Cycling conditions were 35 cycles with annealing temperature 58.5^o^C flowed by cycle sequencing of PCR products with the respective forward and reverse primer using an automated ABI 310 DNA sequencer. 

*Detection of the kozak gene polymorphism*

The following primers (F. 5`: GGG AGT AGG GAG GAC AGG AG. 3` and R 5`: AGT GTA AGG CAT CAG GGT TG. 3`) were used. The annealing temperature was 60oC. The PCR products (348bp) were separated by 2% agarose gel electrophoresis and then digested by Eco471 restriction enzyme (#FD0314) (Thermo Fisher Scientific Inc., Waltham, MA, USA) using PCR-RFLP technique. The restriction products were 129 bp and the 219 bp for the homozygous TT genotype, 348 bp for the homozygous cc genotype and 219 bp, 219 bp and 348 bp for the heterozygous TC genotype. 

*Statistical analysis*

Statistical analysis of collected data was done using statistical package for social science (IBM Corp. Released 2017. IBM SPSS Statistics for Windows Version 25.0. Armonk, NY: IBM Corp, USA). Data were presented and suitable analysis was done according to the type of data obtained for each parameter.

## Results

The present study was conducted on 286 diagnosed patients with Philadelphia-negative MPNs (94 PV patients, 102 ET patients and 90 MF patients). The mean age was 50.2 years, they were 154 males (53.8%) and 132 females (46.2%). In addition, 107 apparently healthy individuals served as a control group of matched age and gender. No significant differences were found in age and gender between PV, ET and MF versus control group. As well as, no significant differences were found in age and gender between PV, ET and MF patients. Regarding the routine laboratory date, It was found that no statistic significant difference between HB, RBCS, WBCS, platelets, INR, PTT and fibrinogen concentration at the level of genotypes TC, CC and TC + CC compared to reference TT genotype in PV, ET and PMF patients (P > 0.05).

When selected patients were followed up for 24 months by radiological investigation using color Doppler ultrasonography, it was found that 74 patients (25.8%) developed thrombosis; 13 (4.54%) at time of diagnosis and 59 (21.33%) during the follow up period. Two patients (0.69%) developed lower limb arterial thrombosis and 72 patients (25.2%) with venous thrombosis; 46 (16.08%) lower limb, 23(8.04%) mesentric vein and 3 (1.04%) upper limb. It occurred in 35 patients (12.2%) with PV, 30 patients (10.48%) with ET and 9 patients (3.15%) with MF.

As regard, the allelic distribution of *Kozak* gene in patients and control groups, taking rs2243093 TT as the reference genotype and T as the reference allele, it was found that TC, CC, TC+CC genotypes and C allele showed lower frequency in ET patients (P = 0.005, 0.007, 0.001 and ≤ 0.001 respectively) and MF patients (p= 0.002, 0.047 0.001 and ≤ 0.001 respectively) when compared to control groups. On the other hand, the CC genotypes and C allele showed only have a lower frequency in PV patients when compared to control groups (p= 0.032 and 0.026 respectively) (Table 1).

Regarding GP1bα genotypes and its allelic distribution the correlation was performed between them and the incidence of thrombosis in patients group. The significant p values were observed only in ET patients TC+CC genotype compared to reference TT genotype (p= 0.025) as well as the alleles (T and C) (p= 0.01). While for PV patients and MF patients it was found that there was insignificant value between thrombosed and unthrombosed patients at the level of genotypes (TC, CC and TC + CC) compared to reference TT genotype and also, at the level of alleles (T and C) (p> 0.05) (Table 2).

The Comparison of GP1bα genotypes and alleles between patients with negative and positive JAK2 mutations patients in the studied groups showed that positive JAK2 mutation was of no significance difference in all genotypes and alleles in PV and ET patients (P > 0.05) while positive JAK2 mutation was significantly associated with TC and CC genotypes (P = 0.005), TC + CC (P = 0.004) and C allele (P = 0.001) these data were showed in Table 3. It was found that there was insignificant value between JAK2 V617F burden allele median at the level of genotypes TC, CC and TC + CC compared to reference TT genotype (48.2 %, 50.1 %, 49.15 % and 49.1 % respectively in PV patients) (P > 0.05), (18.9 %, 19.5 %, 19.2 % and 20.1% respectively in ET patients) (P > 0.05) and (35.0%, 33.7%, 34.35% and 34.1% respectively in PMF patients) (P > 0.05).

As regard CALR mutation, the comparison of GP1bα genotypes and alleles between patients with negative and positive CALR mutations in ET and MF groups was performed and showed no significant association in GP1bα genotypes and alleles between patients with negative and positive CALR mutations (P > 0.05 for all).

Regression analysis was conducted for prediction of PV, ET and MF, using age, gender, GP1bα genotypes, *JAK2* and *CALR* mutations as covariates. GP1bα dominant model was considered as protective predictor for PV, ET and MF in uni- and multivariable analyses. Positive JAK2 in PV, ET and MF, as well as positive CALR in ET and MF were associated with risk of PV, ET and MF respectively in uni- and multivariable analyses (P< 0.001) these data were presented in Table 4.

**Table 1:** Comparison of GP1bα Genotypes and Alleles between PV, ET, MF Patients and Control Groups

rs2243093 Genotypes	Control N=107		PV N=94		ET N=102		MF N=90		P1	OR	95% CI	P2	OR	95% CI	P3	OR	95% CI
	N	%	N	%	N	%	N	%									
TT	52	48.6	58	61.7	73	71.6	65	72.2		1	(reference)		1	(reference)		1	(reference)
TC	46	43	34	36.2	28	27.5	22	24.4	0.164	0.773	0.538-1.111	0.005	0.593	0.412-0.855	0.002	0.55	0.375-0.808
CC	9	8.4	2	2.1	1	1	3	3.3	0.032	0.376	0.154-0.921	0.007	0.225	0.076-0.663	0.047	0.443	0.198-0.990
TC+CC	55	51.4	36	38.3	29	28.4	25	27.8	0.063	0.717	0.505-1.018	0.001	0.543	0.381-0.774	0.001	0.533	0.370-0.769
Alleles																	
T	150	70.1	150	79.8	174	85.3	152	84.4		1	(reference)		1	(reference)		1	(reference)
C	64	29.9	38	20.2	30	14.7	28	15.6	0.026	0.722	0.543-0.961	<0.001	0.569	0.423-0.766	0.001	0.594	0.439-0.805
pHW	0.793		0.239		0.341		0.509										

P1, PV versus control; P2, ET versus control; P3, MF versus control; OR, odds ratio; CI, confidence interval; pHW, p value for Hardy Weinberg equation.

**Table 2:** Correlation between GP1bα Genotypes and Alleles with Incidence Thrombosed and Unthrombosed MPNs Patients

Genotype	PV N=94					ET N=102					MF N=90				
	Unthrombosed N=59		Thrombosed N=35		P	Unthrombosed N=72		Thrombosed N=30		p	Unthrombosed N=81		Thrombosed N=9		p
	N	%	N	%		N	%	N	%		N	%	N	%	
TT	32	54.2	26	74.3	0.131	50	69.4	14	46.7	0.092	56	69.1	9	100	0.161
TC	25	42.4	9	25.7		20	27.8	12	40.0		22	27.2	0	0	
CC	2	3.4	0	0.0		2	2.8	4	13.3		3	3.7	0	0	
TT	32	54.2	26	74.3	0.053	50	69.4	14	46.7	0.025	56	69.1	9	100	0.058
TC+CC	27	45.8	9	25.7		22	30.6	16	53.3		25	30.9	0	0	
Alleles															
T	89	75.4	61	87.1	0.053	120	83.3	40	66.7	0.01	134	82.7	18	100	0.08
C	29	24.6	9	12.9		24	16.7	20	33.3		28	17.3	0	0	

**Table 3:** Comparison of GP1bα Genotypes and Alleles between Patients with Negative and Positive *JAK2* Mutations in PV, ET and MF Groups

Genotype	PV N=94					ET N=102					MF N=90				
	Negative JAK2 N=35		Positive JAK2 N=59		p	Negative JAK2 N=62		Positive JAK2 N=40		p	Negative JAK2 N=33		Positive JAK2 N=57		p

	N	%	N	%		N	%	N	%		N	%	N	%	
TT	26	74.3	32	54.2	0.13	42	67.7	31	77.5	0.616	18	54.5	47	82.5	0.005
TC	9	25.7	25	42.4		19	30.6	9	22.5		12	36.4	10	17.5	
CC	0	0	2	3.4		1	1.6	0	0		3	9.1	0	0	
TT	26	74.3	32	54.2	0.06	42	67.7	31	77.5	0.286	18	54.5	47	82.5	0.004
TC+CC	9	25.7	27	45.8		20	32.3	9	22.5		15	45.5	10	17.5	
Alleles															
T	61	87.1	89	75.4	0.05	103	83.1	71	88.8	0.263	48	72.7	104	91.2	0.001
C	9	12.9	29	24.6		21	16.9	9	11.3		18	27.3	10	8.8	

**Table 4:** Regression Analysis for Prediction of PV, ET and MF

		Univariable			Multivariable		
		P	OR	95% CI	P	OR	95% CI
Prediction of PV	Age	0.889	0.999	0.980-1.018			
	Gender	0.431	0.792	0.443-1.415			
	TC+CC	0.049	0.717	0.505-0.918	0.034	0.754	0.498-0.943
	Positive JAK2	<0.001	4.139	3.232-5.302	<0.001	3.957	2.605-6.012
Prediction of ET	Age	0.14	0.989	0.779-1.498			
	Gender	0.342	2.316	0.627-3.295			
	TC+CC	0.001	0.543	0.381-0.774	0.038	0.562	0.303-0.916
	Positive JAK2	<0.001	2.644	1.996-3.502	<0.001	6.029	4.081-8.907
	Positive CALR	<0.001	2.702	2.045-3.571	<0.001	6.045	4.114-8.882
Prediction of MF	Age	0.568	0.996	0.984-1.009			
	Gender	0.373	0.846	0.586-1.222			
	TC+CC	0.001	0.533	0.370-0.769	0.025	0.699	0.224-0.966
	Positive JAK2	<0.001	4.139	3.232-5.302	<0.001	5.469	4.070-7.349
	Positive CALR	<0.001	2.078	1.396-3.092	<0.001	5.044	3.182-7.997

OR, odds ratio; CI, confidence interval

## Discussion

Philadelphia-negative MPNs (MPNs) are stem cell disorder which characterized by clonal dysregulated myeloproliferation and consists of polycythemia vera (PV), essential thrombocythemia (ET), and Myelofibrosis (MF) where the significant causes of morbidity and mortality in those patients are thromboembolic events (Seguro et al., 2020).

Glycoprotein Ib-IX-V is a platelet membrane receptor complex that participates greatly in platelet activity and thrombosis. The glycoprotein Ib-IX-V complex contains four polypeptides, GPIbα, GPIbB, GP IX, and GP V. The biggest subunit is GPIbα which contains all of the known extra cellular ligand-binding sites (Uff et al., 2002).

There are four polymorphisms related to GPIbα region. The Kozak sequence polymorphism which contains a single nucleotide substitution is considered highly correlated with thrombotic and ischemic events (Meisel et al., 2001).

This study aimed to evaluate the predictive value of *Kozak* gene polymorphism for thrombosis in patients with Philadelphia-negative MPNs.

Present study included 286 diagnosed patients with Philadelphia-negative MPNs (94 patients of PV, 102 of ET and 90 of MF). In addition, 107 apparently healthy individuals were subjected as a control group. 

Radiological investigation using color Doppler ultrasonography showed that 74 patients (25.8%) developed thrombosis; 13 (4.54%) at time of diagnosis and 59 (21.33%) during the follow up period 24 months. It occurred in 35 patients (12.2%) with PV, 30 patients (10.48%) with ET and 9 patients (3.15%) with MF. Only 2 patients (0.69%) developed lower limb arterial thrombosis and 72 patients (25.2%) with venous thrombosis; 46 (16.08%) LLs, 23(8.04%) mesentric vein and 3 (1.04%) ULs. 

Mattar et al., (2019) reported that color Doppler ultrasonography of 73 patients with Philadelphia-negative MPNs revealed venous thrombosis at different sites; LLs (11.4%), mesenteric vein (28.6%) and IVC (2.8%) detected in 42.8% of patients with PV, 31.8% of patients with ET and in 25% of patients with MF. In contrast, Di Veroli et al., (2016) studied the incidence of thrombosis in patients with Philadelphia-Negative MPNs and reported 7 venous episodes of thrombosis in 1,087 patients during a median follow-up period of 18 months.

In the present study, we found that TC, CC, TC+CC genotypes showed significant lower frequency in ET patients (p= 0.005, 0.007 and 0.001 respectively) and MF patients (p= 0.002, 0.047 and 0.001 respectively) when compared to control groups. Also, C allele in both groups compared to control (p ≤ 0.001 both). Also, the CC genotype and C allele showed lower frequency in PV patients when compared to control groups (p= 0.032) and (p= 0.026).

As regard GP1bα genotypes and alleles, the correlation was performed between them and the incidence of thrombosed and unthrombosed MPNs patients. For PV patients and MF patients it was found that there was insignificant value between thrombosed and unthrombosed patients at the level of genotype (TC, CC and TC + CC) also, at the level of alleles (T and C) (p> 0.05). For ET there was a significant value observed for only TC+CC genotype compared to reference TT genotype (p= 0.025) as well as the alleles (T and C) (p= 0.01)

Hsieh et al., (2004) using data from the Vienna Stroke Registry found that patients who were homozygous for the CC genotype had a 3.5-fold increased risk for ischemic cerebrovascular events (P = 0.0003) compared with the TT or TC genotype carriers. Streifler et al., (2001) investigated whether the development of stroke or transient ischemic attack in patients with significant carotid stenosis was associated with several platelet glycoprotein polymorphisms. They compared symptomatic and asymptomatic patients and found no significant differences in the frequency of the Kozak sequence polymorphism between the 2 groups. 

The platelet glycoprotein (GP) Ib-IX-V receptor complex, comprising four polypeptides, plays a crucial role in this process by mediating platelet adhesion by binding von Willebrand factor (vWF) at the site of the vessel wall lesion. The T/C polymorphism in the Kozak sequence of GP Ibα (the vWF-binding subunit of the complex) at position -5 from the initiator ATG demonstrated increased GP Ib-IX-V complex density in carriers of the C allele. A possible explanation for this observation could be more efficient messenger ribonucleic acid translation of the -5 C allele, due to closer approximation of the consensus nucleotide sequence derived by Kozak. So, it could be hypothesized that a higher GPIbα receptor density may predispose to thrombotic events (Maguire et al., 2008).

The Comparison of GP1bα genotypes and alleles between patients with negative and positive JAK2 mutations in all studied groups showed that positive JAK2 mutation was of no significance difference in all genotypes and alleles of PV and also ET patients (p > 0.05) while positive JAK2 mutation was significantly associated with TT genotype, TC, CC (p = 0.005) TC + CC (p = 0.004) also T allele and C allele (p= 0.001) in MF patients. The regression analysis was conducted for prediction of PV, ET and MF, using age, gender, GP1bα genotypes, JAK2 and CALR mutations as covariates. GP1bα dominant model was considered as protective predictor for PV, ET and MF in uni- and multivariable analyses. Positive JAK2 in PV, ET and MF, as well as positive CALR in ET and MF were associated with risk of PV, ET and MF respectively in uni- and multivariable analyses (P< 0.001).

The study of Borowczyk et al., (2015) documented that in patients with Philadelphia-negative MPNs, the presence of JAK2V617F mutation and the allele burden were shown to be related to the risk of venous thrombosis. Patients harboring JAK2 V617F mutation were at higher risk of VTE (P = 0.024), mainly deep vein thrombosis (DVT). JAK2 allele burden higher than 20% identified patients with 7.4-fold increased risk of VTE (P = 0.004). 

Horvat et al., (2019) performed a cohort study on 258 patients with Philadelphia-negative MPNs, 79 (30.6%) had a total of 109 thrombotic events, 66.1% arterial and 33.9% venous. Moreover, their results demonstrated that the incidence of venous thrombosis in 11.4% of PV patients, 7.5% of ET patients and MF patients showed much higher incidence of thrombosis 35.2%. PV patients with V617F burden allele > 90.4% were more prone to venous thrombosis (P = 0.036), ET patients with V617F burden allele > 18.3% with insignificant incidence of venous thrombosis (P = 0.28) In the group of MF patients, only V617F burden allele > 56.7% was more often observed in patients with venous thrombosis (P = 0.046).

The authors of the IPSET scoring system also recognized the presence of the V617F mutation as one of contributing factors to thrombosis (Barbui et al., 2012).

Although the presence of this mutation is much higher in PV than in ET and MF patients, some studies showed that additional information can be obtained from quantifying V617F burden allele in each of the MPNs subgroups (Antonioli et al., 2008).

Conclusion: From this study we could conclude that patients with Philadelphia-negative MPNs carrying *Kozak* gene polymorphism significantly TT genotype in all patients PV, ET, MF patients and TC in ET and MF patients. The platelet glycoprotein Ibα (*Kozak*) gene could be incorporated into the routine workup to predict venous thrombosis in patients with Philadelphia-negative MPNs specially ET patients as it’s easily determined by PCR-RFLP. Also, targeting this polymorphism may help in development of antithrombotic drugs in those patients.

## Author Contribution Statement

The authors confirm contribution to the paper as follows: study conception and design: Mohamed Sabry El-Ghonemy and Ahmed EL-Sebaie; data collection and analysis and interpretation of results:

Mohamed Sabry El-Ghonemy , Shaimaa El-Ashwah, May Denewer, Eman Adel Soliman, El-Baiomy MA, Heidi Elkerdawy; draft manuscript preparation: Ahmed EL-Sebaie. All authors reviewed the results and approved the final version of the manuscript.
